# Tris(naphthalen-1-yl)phosphane chloro­form hemisolvate

**DOI:** 10.1107/S1600536812048234

**Published:** 2012-11-30

**Authors:** Wade L. Davis, Alfred Muller

**Affiliations:** a Research Centre for Synthesis and Catalysis, Department of Chemistry, University of Johannesburg (APK Campus), PO Box 524, Auckland Park, Johannesburg, 2006, South Africa

## Abstract

The title compound, P(C_10_H_7_)_3_·0.5CHCl_3_, was isolated after the unsuccessful reaction of KSeCN and tris­(naphthalen-1-yl)phosphane. The solvent mol­ecule is disordered about an inversion center. The effective cone angle of the phosphine is 203°. In the crystal, weak C—H⋯Cl and C—H⋯π inter­actions are observed.

## Related literature

For background to the investigation of the steric and electronic properties of phospho­rus-containing ligands, see: Otto & Roodt (2004 ▶); Cowley & Damasco (1971 ▶); Allen & Taylor (1982 ▶); Allen *et al.* (1985 ▶); Muller *et al.* (2008 ▶). For background to cone angles, see: Tolman (1977 ▶); Otto (2001 ▶).
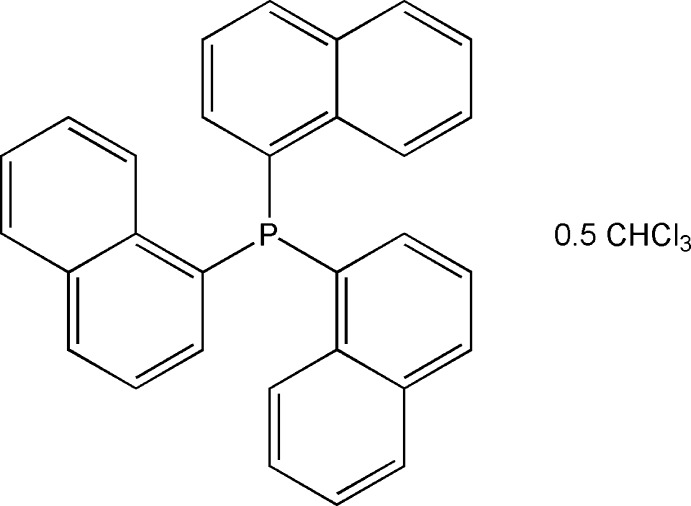


## Experimental

### 

#### Crystal data

C_30_H_21_P·0.5CHCl_3_
*M* *_r_* = 472.12
Monoclinic, 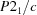

*a* = 9.197 (3) Å
*b* = 14.564 (5) Å
*c* = 18.675 (5) Å
β = 107.061 (14)°
*V* = 2391.3 (13) Å^3^
*Z* = 4
Mo *K*α radiation
μ = 0.3 mm^−1^
*T* = 100 K
0.3 × 0.07 × 0.07 mm

#### Data collection

Bruker APEX DUO 4K-CCD diffractometer
Absorption correction: multi-scan (*SADABS*; Bruker, 2008 ▶) *T*_min_ = 0.916, *T*_max_ = 0.979
23695 measured reflections
5950 independent reflections
3713 reflections with *I* > 2σ(*I*)
*R*_int_ = 0.107

#### Refinement

*R*[*F* ^2^ > 2σ(*F* ^2^)] = 0.066
*wR*(*F* ^2^) = 0.194
*S* = 1.02
5950 reflections
316 parameters
H-atom parameters constrained
Δρ_max_ = 0.87 e Å^−3^
Δρ_min_ = −0.57 e Å^−3^

### 

Data collection: *APEX2* (Bruker, 2011 ▶); cell refinement: *SAINT* (Bruker, 2008 ▶); data reduction: *SAINT* and *XPREP* (Bruker, 2008 ▶); program(s) used to solve structure: *SIR97* (Altomare *et al.*, 1999 ▶); program(s) used to refine structure: *SHELXL97* (Sheldrick, 2008 ▶); molecular graphics: *DIAMOND* (Brandenburg & Putz, 2005 ▶); software used to prepare material for publication: *publCIF* (Westrip, 2010 ▶) and *WinGX* (Farrugia, 2012 ▶).

## Supplementary Material

Crystal structure: contains datablock(s) global, I. DOI: 10.1107/S1600536812048234/lh5561sup1.cif

Structure factors: contains datablock(s) I. DOI: 10.1107/S1600536812048234/lh5561Isup2.hkl

Supplementary material file. DOI: 10.1107/S1600536812048234/lh5561Isup3.cml

Additional supplementary materials:  crystallographic information; 3D view; checkCIF report

## Figures and Tables

**Table 1:** Hydrogen-bond geometry (Å, °) *Cg*1, *Cg*2, *Cg*3 and *Cg*4 are the centroids of the C2–C7, C12–C17, C25–C30 and C1/C2/C7–C10 rings, respectively.

*D*—H⋯*A*	*D*—H	H⋯*A*	*D*⋯*A*	*D*—H⋯*A*
C5—H5⋯Cl1^i^	0.93	2.82	3.512 (4)	132
C18—H18⋯*Cg*1^ii^	0.93	2.66	3.579 (3)	170
C24—H24⋯*Cg*2^iii^	0.93	2.51	3.425 (3)	167
C27—H27⋯*Cg*2^iv^	0.93	2.69	3.612 (3)	170
C8—H8⋯*Cg*3^v^	0.93	2.79	3.580 (3)	143
C31—H31⋯*Cg*4^vi^	0.98	2.65	3.618 (6)	172

Symmetry codes: (i) 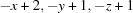; (ii) 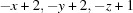; (iii) 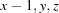; (iv) 
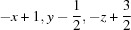
; (v) 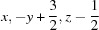; (vi) 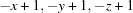.

**Fig. 1.**
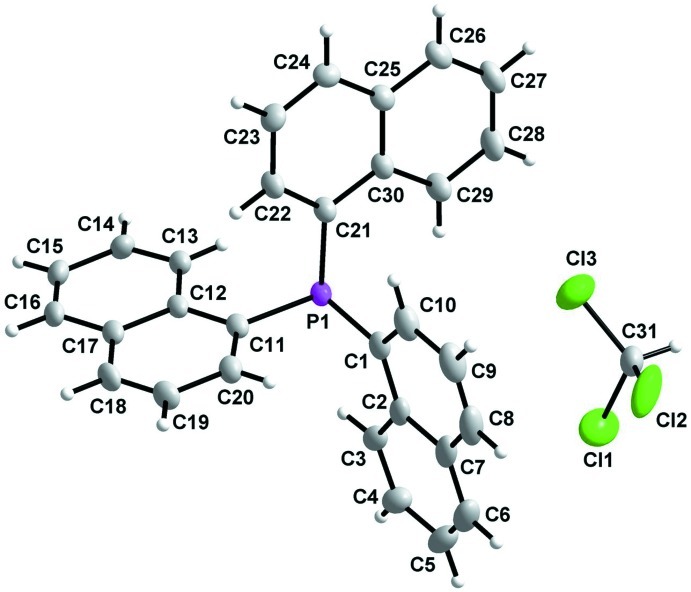
A view of the title complex, showing 50% probability displacement ellipsoids. The chloroform solvent molecule is half occupancy.

**Fig. 2.**
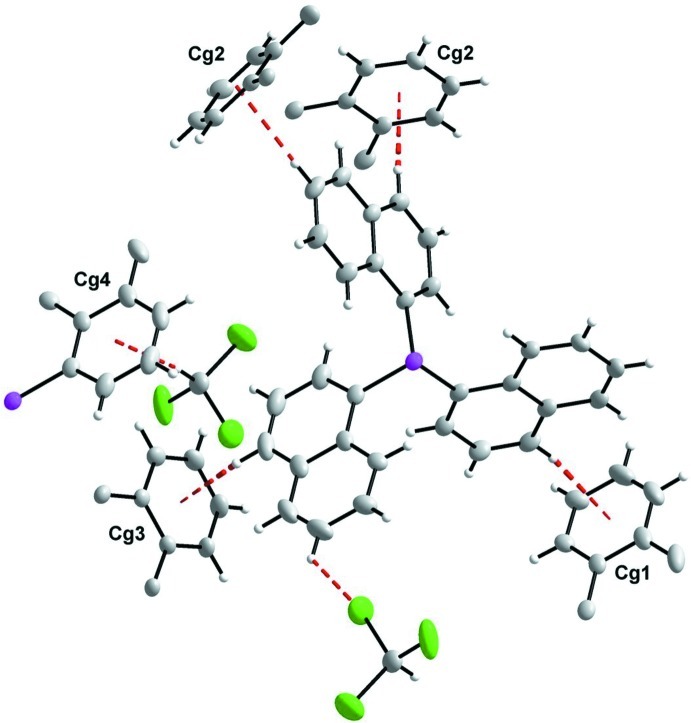
Packing diagram showing the C—H···Cl/π interactions (indicated by red dashed lines).

## supplementary crystallographic information

### Comment

Several techniques to rapidly evaluate steric and electronic properties of phoshane ligands have been developed over the past few decades. Highlights from these studies include the measuring of IR stretching frequencies in complexes such as [NiP(CO)_3_] (Tolman, 1977), *trans*-[RhCl(CO)(P)_2_] (Otto & Roodt, 2004) and by the measuring of coupling constants between ^31^P and other NMR active nuclei such as ^11^B, ^195^Pt or ^77^Se (Cowley & Damasco, 1971; Allen & Taylor, 1982; Allen *et al.*, 1985). In our research into these properties we make use of seledized phosphane ligands, providing useful probes such as ^1^*J*(^31^P-^77^Se) coupling, Se—P bond distance and kinetic reaction rates (Muller *et al.*, 2008). The title compound (Fig. 1) in the present study was obtained during an unsuccessful reaction between KSeCN and tris(naphthalen-1-yl)phoshane in MeOH:CHCl_3_ (1:1).

The molecular structure of the title compound is shown in Fig. 1. The chloroform solvent molecule is disordered across an inversion center. The average P–C distance and C–P–C angle are 1.837 (3) Å and 102.43 (12)°, respectively. To describe the steric demand of the phosphane ligands the Tolman cone angle (Tolman, 1977) is still the most commonly used model. Applying this model to the geometry obtained from the title compound with a dummy atom positioned at a distance of 2.28 Å from the P-atom, we calculated an effective cone angle (Otto, 2001) of 203°. This large value may account for the unreactiveness of the phosphorus centre with selenium.

Packing in the crystals is assisted by weak C—H···Cl and C—H···π interactions (see table 1 and Fig. 2 for a graphical representation of these interactions).

### Experimental

Tris(naphthalen-1-yl)phosphane and KSeCN were purchased from Sigma-Aldrich and used without purification. Eqimolar amounts of KSeCN (5.8 mg, 0.04 mmol) and tris(naphthalen-1-yl)phosphane (16.5 mg, 0.04 mmol) were dissolved in the minimum amount of methanol (5 ml) and chloroform (5 ml), respectively. The KSeCN solution was added drop wise (5 min.) to the phosphane solution with stirring at room temperature (1hr.). Slow evaporation of the solvent afforded the title compound as colourless needles suitable for a single-crystal X-ray study. Analytical data: ^31^P {H} NMR (CDCl_3_, 161.99 MHz): δ = -33.15 (s, 1P)

### Refinement

The aromatic and methine H atoms were placed in geometrically idealized positions (C—H = 0.93 and 0.98) Å and allowed to ride on their parent atoms, with *U*_iso_(H) = 1.2*U*_eq_(C). The chloroform solvate molecule is disordered across an inversion centre, H atom connectivity was correctly assigned by using a PART -1 instruction in SHELXL-97 (Sheldrick, 2008). Occupancies of each disordered component were constrained to 50% conforming to the imposed crystallographic symmetry. No additional geometrical or thermal ellipsoid restrains were employed in the final refinement cycles.

### Crystal data

**Table:**

C_30_H_21_P·0.5CHCl_3_	*F*(000) = 980
*M**_r_* = 472.12	*D*_x_ = 1.311 Mg m^−^^3^
Monoclinic, *P*2_1_/*c*	Mo *K*α radiation, λ = 0.71073 Å
Hall symbol: -P 2ybc	Cell parameters from 2813 reflections
*a* = 9.197 (3) Å	θ = 2.3–24.3°
*b* = 14.564 (5) Å	µ = 0.3 mm^−^^1^
*c* = 18.675 (5) Å	*T* = 100 K
β = 107.061 (14)°	Needle, colourless
*V* = 2391.3 (13) Å^3^	0.3 × 0.07 × 0.07 mm
*Z* = 4	

### Data collection

**Table:**

Bruker APEX DUO 4K-CCD diffractometer	5950 independent reflections
Radiation source: sealed tube	3713 reflections with *I* > 2σ(*I*)
Graphite monochromator	*R*_int_ = 0.107
Detector resolution: 8.4 pixels mm^-1^	θ_max_ = 28.4°, θ_min_ = 1.8°
φ and ω scans	*h* = −12→12
Absorption correction: multi-scan (*SADABS*; Bruker, 2008)	*k* = −18→19
*T*_min_ = 0.916, *T*_max_ = 0.979	*l* = −24→24
23695 measured reflections	

### Refinement

**Table:**

Refinement on *F*^2^	Primary atom site location: structure-invariant direct methods
Least-squares matrix: full	Secondary atom site location: difference Fourier map
*R*[*F*^2^ > 2σ(*F*^2^)] = 0.066	Hydrogen site location: inferred from neighbouring sites
*wR*(*F*^2^) = 0.194	H-atom parameters constrained
*S* = 1.02	*w* = 1/[σ^2^(*F*_o_^2^) + (0.1042*P*)^2^ + 0.0263*P*] where *P* = (*F*_o_^2^ + 2*F*_c_^2^)/3
5950 reflections	(Δ/σ)_max_ < 0.001
316 parameters	Δρ_max_ = 0.87 e Å^−^^3^
0 restraints	Δρ_min_ = −0.57 e Å^−^^3^

### Special details

**Table:**

Experimental. The intensity data was collected on a Bruker Apex DUO 4 K CCD diffractometer using an exposure time of 120 s/frame. A total of 1041 frames were collected with a frame width of 0.5° covering up to θ = 28.38° with 99.2% completeness accomplished.
Geometry. All e.s.d.'s (except the e.s.d. in the dihedral angle between two l.s. planes) are estimated using the full covariance matrix. The cell e.s.d.'s are taken into account individually in the estimation of e.s.d.'s in distances, angles and torsion angles; correlations between e.s.d.'s in cell parameters are only used when they are defined by crystal symmetry. An approximate (isotropic) treatment of cell e.s.d.'s is used for estimating e.s.d.'s involving l.s. planes.
Refinement. Refinement of *F*^2^ against ALL reflections. The weighted *R*-factor *wR* and goodness of fit *S* are based on *F*^2^, conventional *R*-factors *R* are based on *F*, with *F* set to zero for negative *F*^2^. The threshold expression of *F*^2^ > σ(*F*^2^) is used only for calculating *R*-factors(gt) *etc*. and is not relevant to the choice of reflections for refinement. *R*-factors based on *F*^2^ are statistically about twice as large as those based on *F*, and *R*- factors based on ALL data will be even larger.

### Fractional atomic coordinates and isotropic or equivalent isotropic displacement parameters (Å^2^)

**Table:**

	*x*	*y*	*z*	*U*_iso_*/*U*_eq_	Occ. (<1)
P1	0.73007 (7)	0.85270 (5)	0.62816 (4)	0.02204 (18)	
C1	0.6589 (3)	0.80104 (19)	0.53493 (14)	0.0251 (5)	
C2	0.7611 (3)	0.74531 (18)	0.50785 (15)	0.0269 (6)	
C3	0.9148 (3)	0.72986 (19)	0.54983 (15)	0.0277 (6)	
H3	0.9534	0.7578	0.5963	0.033*	
C4	1.0071 (4)	0.6744 (2)	0.52300 (17)	0.0373 (7)	
H4	1.1069	0.664	0.5518	0.045*	
C5	0.9517 (5)	0.6332 (2)	0.4522 (2)	0.0502 (9)	
H5	1.0149	0.5956	0.4343	0.06*	
C6	0.8055 (4)	0.6484 (2)	0.40970 (18)	0.0474 (9)	
H6	0.7708	0.6216	0.3625	0.057*	
C7	0.7056 (4)	0.7039 (2)	0.43570 (16)	0.0353 (7)	
C8	0.5525 (4)	0.7185 (2)	0.39308 (17)	0.0439 (8)	
H8	0.5166	0.6925	0.3457	0.053*	
C9	0.4568 (4)	0.7699 (2)	0.42033 (18)	0.0427 (8)	
H9	0.3559	0.7775	0.3921	0.051*	
C10	0.5105 (3)	0.8114 (2)	0.49096 (17)	0.0354 (7)	
H10	0.4443	0.8469	0.5086	0.042*	
C11	0.8153 (3)	0.95984 (18)	0.60744 (15)	0.0243 (5)	
C12	0.9083 (3)	1.01264 (17)	0.66864 (15)	0.0237 (5)	
C13	0.9367 (3)	0.98622 (19)	0.74493 (15)	0.0265 (6)	
H13	0.8914	0.9334	0.7565	0.032*	
C14	1.0293 (3)	1.0370 (2)	0.80119 (16)	0.0312 (6)	
H14	1.0476	1.0181	0.8506	0.037*	
C15	1.0980 (3)	1.1185 (2)	0.78521 (17)	0.0325 (6)	
H15	1.1608	1.1529	0.824	0.039*	
C16	1.0719 (3)	1.14612 (19)	0.71339 (16)	0.0302 (6)	
H16	1.1168	1.2	0.7035	0.036*	
C17	0.9776 (3)	1.09469 (18)	0.65280 (16)	0.0262 (6)	
C18	0.9538 (3)	1.12187 (19)	0.57754 (17)	0.0316 (6)	
H18	0.999	1.1754	0.5672	0.038*	
C19	0.8659 (3)	1.0710 (2)	0.52010 (16)	0.0307 (6)	
H19	0.8516	1.0895	0.4709	0.037*	
C20	0.7961 (3)	0.98966 (19)	0.53533 (16)	0.0281 (6)	
H20	0.7357	0.9555	0.4956	0.034*	
C21	0.5560 (3)	0.89395 (18)	0.64718 (14)	0.0238 (5)	
C22	0.5185 (3)	0.98566 (19)	0.64516 (15)	0.0270 (6)	
H22	0.5778	1.028	0.6289	0.032*	
C23	0.3931 (3)	1.01723 (19)	0.66694 (15)	0.0293 (6)	
H23	0.3705	1.0796	0.6647	0.035*	
C24	0.3042 (3)	0.9567 (2)	0.69129 (15)	0.0269 (6)	
H24	0.2224	0.9783	0.7061	0.032*	
C25	0.3358 (3)	0.86131 (19)	0.69407 (14)	0.0248 (5)	
C26	0.2434 (3)	0.7973 (2)	0.71761 (15)	0.0302 (6)	
H26	0.1604	0.8183	0.7318	0.036*	
C27	0.2735 (3)	0.7053 (2)	0.71984 (16)	0.0344 (7)	
H27	0.212	0.6642	0.7357	0.041*	
C28	0.3985 (3)	0.6730 (2)	0.69789 (17)	0.0345 (7)	
H28	0.4186	0.6103	0.699	0.041*	
C29	0.4911 (3)	0.73308 (19)	0.67483 (16)	0.0297 (6)	
H29	0.5735	0.7105	0.661	0.036*	
C30	0.4632 (3)	0.82826 (18)	0.67181 (15)	0.0248 (6)	
Cl1	0.68271 (18)	0.46375 (16)	0.50033 (11)	0.0583 (5)	0.5
Cl2	0.3885 (2)	0.49626 (13)	0.39948 (12)	0.0646 (6)	0.5
Cl3	0.4575 (3)	0.55581 (16)	0.55292 (13)	0.0727 (6)	0.5
C31	0.4885 (7)	0.4716 (4)	0.4923 (4)	0.0397 (14)	0.5
H31	0.4538	0.4123	0.5059	0.048*	0.5

### Atomic displacement parameters (Å^2^)

**Table:**

	*U*^11^	*U*^22^	*U*^33^	*U*^12^	*U*^13^	*U*^23^
P1	0.0227 (3)	0.0153 (3)	0.0283 (4)	0.0009 (2)	0.0078 (3)	0.0026 (3)
C1	0.0304 (13)	0.0181 (13)	0.0257 (13)	−0.0033 (10)	0.0064 (11)	0.0056 (11)
C2	0.0379 (14)	0.0167 (13)	0.0263 (14)	−0.0059 (11)	0.0100 (12)	0.0026 (11)
C3	0.0392 (14)	0.0205 (14)	0.0267 (14)	−0.0006 (11)	0.0149 (12)	0.0035 (11)
C4	0.0479 (17)	0.0290 (16)	0.0412 (17)	0.0038 (13)	0.0229 (14)	0.0015 (14)
C5	0.080 (3)	0.0308 (19)	0.055 (2)	−0.0040 (16)	0.042 (2)	−0.0103 (16)
C6	0.078 (2)	0.0366 (19)	0.0331 (17)	−0.0182 (17)	0.0254 (17)	−0.0112 (15)
C7	0.0557 (18)	0.0221 (15)	0.0292 (15)	−0.0128 (13)	0.0143 (14)	0.0002 (12)
C8	0.061 (2)	0.0358 (19)	0.0274 (16)	−0.0208 (15)	0.0018 (15)	0.0008 (14)
C9	0.0409 (16)	0.041 (2)	0.0363 (17)	−0.0134 (14)	−0.0040 (14)	0.0095 (15)
C10	0.0340 (14)	0.0288 (17)	0.0368 (16)	−0.0055 (12)	0.0002 (13)	0.0099 (13)
C11	0.0214 (11)	0.0177 (13)	0.0351 (15)	0.0024 (9)	0.0102 (11)	0.0032 (11)
C12	0.0231 (11)	0.0154 (12)	0.0354 (15)	0.0014 (9)	0.0129 (11)	0.0014 (11)
C13	0.0302 (13)	0.0179 (13)	0.0351 (15)	−0.0013 (10)	0.0153 (12)	−0.0001 (11)
C14	0.0419 (15)	0.0260 (15)	0.0305 (15)	−0.0044 (12)	0.0180 (13)	−0.0054 (12)
C15	0.0361 (14)	0.0226 (15)	0.0427 (17)	−0.0059 (11)	0.0178 (13)	−0.0103 (13)
C16	0.0338 (13)	0.0159 (13)	0.0458 (17)	−0.0035 (10)	0.0196 (13)	−0.0045 (12)
C17	0.0265 (12)	0.0151 (13)	0.0402 (16)	0.0015 (10)	0.0149 (11)	0.0007 (11)
C18	0.0345 (14)	0.0175 (14)	0.0468 (17)	−0.0016 (10)	0.0181 (13)	0.0070 (12)
C19	0.0337 (14)	0.0243 (15)	0.0349 (16)	0.0014 (11)	0.0115 (12)	0.0108 (12)
C20	0.0278 (13)	0.0205 (14)	0.0356 (15)	0.0008 (10)	0.0088 (12)	0.0027 (12)
C21	0.0217 (11)	0.0187 (13)	0.0306 (14)	0.0016 (9)	0.0071 (11)	0.0045 (11)
C22	0.0256 (12)	0.0181 (13)	0.0375 (16)	0.0014 (10)	0.0098 (12)	0.0068 (11)
C23	0.0306 (14)	0.0201 (14)	0.0362 (16)	0.0056 (11)	0.0081 (12)	0.0019 (12)
C24	0.0222 (12)	0.0290 (15)	0.0291 (14)	0.0048 (10)	0.0068 (11)	0.0019 (12)
C25	0.0204 (11)	0.0268 (14)	0.0252 (13)	0.0000 (10)	0.0036 (10)	0.0039 (11)
C26	0.0220 (12)	0.0346 (16)	0.0323 (15)	−0.0031 (11)	0.0053 (11)	0.0049 (13)
C27	0.0294 (13)	0.0329 (16)	0.0393 (17)	−0.0092 (12)	0.0075 (12)	0.0091 (13)
C28	0.0334 (14)	0.0202 (14)	0.0476 (18)	−0.0021 (11)	0.0081 (13)	0.0086 (13)
C29	0.0275 (13)	0.0212 (14)	0.0410 (17)	0.0033 (10)	0.0110 (12)	0.0095 (12)
C30	0.0246 (12)	0.0206 (14)	0.0286 (14)	−0.0009 (10)	0.0069 (11)	0.0038 (11)
Cl1	0.0349 (8)	0.0726 (14)	0.0590 (12)	0.0079 (8)	0.0009 (8)	−0.0100 (10)
Cl2	0.0631 (11)	0.0349 (10)	0.0680 (13)	0.0079 (8)	−0.0239 (10)	−0.0142 (9)
Cl3	0.0889 (15)	0.0602 (14)	0.0794 (15)	0.0005 (11)	0.0409 (13)	−0.0266 (12)
C31	0.046 (3)	0.026 (3)	0.051 (4)	−0.009 (3)	0.020 (3)	−0.008 (3)

### Geometric parameters (Å, º)

**Table:**

P1—C1	1.832 (3)	C16—C17	1.420 (4)
P1—C11	1.838 (3)	C16—H16	0.93
P1—C21	1.840 (2)	C17—C18	1.414 (4)
C1—C10	1.379 (4)	C18—C19	1.358 (4)
C1—C2	1.441 (4)	C18—H18	0.93
C2—C3	1.419 (4)	C19—C20	1.415 (4)
C2—C7	1.427 (4)	C19—H19	0.93
C3—C4	1.369 (4)	C20—H20	0.93
C3—H3	0.93	C21—C22	1.377 (4)
C4—C5	1.404 (5)	C21—C30	1.444 (3)
C4—H4	0.93	C22—C23	1.408 (3)
C5—C6	1.364 (5)	C22—H22	0.93
C5—H5	0.93	C23—C24	1.368 (4)
C6—C7	1.413 (5)	C23—H23	0.93
C6—H6	0.93	C24—C25	1.417 (4)
C7—C8	1.415 (5)	C24—H24	0.93
C8—C9	1.363 (5)	C25—C26	1.416 (4)
C8—H8	0.93	C25—C30	1.436 (3)
C9—C10	1.403 (4)	C26—C27	1.367 (4)
C9—H9	0.93	C26—H26	0.93
C10—H10	0.93	C27—C28	1.410 (4)
C11—C20	1.376 (4)	C27—H27	0.93
C11—C12	1.434 (4)	C28—C29	1.375 (4)
C12—C13	1.424 (4)	C28—H28	0.93
C12—C17	1.426 (4)	C29—C30	1.408 (4)
C13—C14	1.360 (4)	C29—H29	0.93
C13—H13	0.93	Cl1—C31	1.752 (7)
C14—C15	1.416 (4)	Cl2—C31	1.745 (8)
C14—H14	0.93	Cl3—C31	1.748 (7)
C15—C16	1.353 (4)	C31—H31	0.98
C15—H15	0.93		
			
C1—P1—C11	101.78 (12)	C17—C16—H16	119.3
C1—P1—C21	103.24 (12)	C18—C17—C16	121.6 (2)
C11—P1—C21	102.27 (12)	C18—C17—C12	119.5 (3)
C10—C1—C2	119.1 (3)	C16—C17—C12	118.8 (2)
C10—C1—P1	122.4 (2)	C19—C18—C17	121.0 (3)
C2—C1—P1	118.48 (19)	C19—C18—H18	119.5
C3—C2—C7	118.5 (3)	C17—C18—H18	119.5
C3—C2—C1	122.8 (2)	C18—C19—C20	119.9 (3)
C7—C2—C1	118.7 (2)	C18—C19—H19	120.1
C4—C3—C2	121.0 (3)	C20—C19—H19	120.1
C4—C3—H3	119.5	C11—C20—C19	121.7 (3)
C2—C3—H3	119.5	C11—C20—H20	119.2
C3—C4—C5	120.3 (3)	C19—C20—H20	119.2
C3—C4—H4	119.9	C22—C21—C30	119.0 (2)
C5—C4—H4	119.9	C22—C21—P1	122.43 (19)
C6—C5—C4	120.1 (3)	C30—C21—P1	118.32 (19)
C6—C5—H5	119.9	C21—C22—C23	121.9 (2)
C4—C5—H5	119.9	C21—C22—H22	119.1
C5—C6—C7	121.5 (3)	C23—C22—H22	119.1
C5—C6—H6	119.3	C24—C23—C22	120.4 (3)
C7—C6—H6	119.3	C24—C23—H23	119.8
C6—C7—C8	122.2 (3)	C22—C23—H23	119.8
C6—C7—C2	118.6 (3)	C23—C24—C25	120.5 (2)
C8—C7—C2	119.3 (3)	C23—C24—H24	119.7
C9—C8—C7	121.1 (3)	C25—C24—H24	119.7
C9—C8—H8	119.4	C26—C25—C24	121.4 (2)
C7—C8—H8	119.4	C26—C25—C30	119.0 (3)
C8—C9—C10	120.0 (3)	C24—C25—C30	119.6 (2)
C8—C9—H9	120	C27—C26—C25	121.3 (2)
C10—C9—H9	120	C27—C26—H26	119.3
C1—C10—C9	121.8 (3)	C25—C26—H26	119.3
C1—C10—H10	119.1	C26—C27—C28	119.6 (3)
C9—C10—H10	119.1	C26—C27—H27	120.2
C20—C11—C12	119.1 (2)	C28—C27—H27	120.2
C20—C11—P1	122.3 (2)	C29—C28—C27	120.7 (3)
C12—C11—P1	118.63 (19)	C29—C28—H28	119.6
C13—C12—C17	118.2 (2)	C27—C28—H28	119.6
C13—C12—C11	123.0 (2)	C28—C29—C30	121.1 (2)
C17—C12—C11	118.9 (2)	C28—C29—H29	119.4
C14—C13—C12	121.0 (2)	C30—C29—H29	119.4
C14—C13—H13	119.5	C29—C30—C25	118.2 (2)
C12—C13—H13	119.5	C29—C30—C21	123.2 (2)
C13—C14—C15	120.6 (3)	C25—C30—C21	118.6 (2)
C13—C14—H14	119.7	Cl2—C31—Cl3	111.1 (4)
C15—C14—H14	119.7	Cl2—C31—Cl1	109.0 (4)
C16—C15—C14	119.9 (3)	Cl3—C31—Cl1	110.3 (3)
C16—C15—H15	120.1	Cl2—C31—H31	108.8
C14—C15—H15	120.1	Cl3—C31—H31	108.8
C15—C16—C17	121.5 (3)	Cl1—C31—H31	108.8
C15—C16—H16	119.3		
			
C11—P1—C1—C10	−95.6 (2)	C15—C16—C17—C18	−178.1 (3)
C21—P1—C1—C10	10.2 (3)	C15—C16—C17—C12	0.5 (4)
C11—P1—C1—C2	86.3 (2)	C13—C12—C17—C18	178.9 (2)
C21—P1—C1—C2	−167.9 (2)	C11—C12—C17—C18	−0.3 (3)
C10—C1—C2—C3	−179.1 (3)	C13—C12—C17—C16	0.2 (3)
P1—C1—C2—C3	−0.9 (3)	C11—C12—C17—C16	−178.9 (2)
C10—C1—C2—C7	1.0 (4)	C16—C17—C18—C19	178.6 (2)
P1—C1—C2—C7	179.2 (2)	C12—C17—C18—C19	0.0 (4)
C7—C2—C3—C4	−1.9 (4)	C17—C18—C19—C20	0.3 (4)
C1—C2—C3—C4	178.2 (3)	C12—C11—C20—C19	0.0 (4)
C2—C3—C4—C5	1.4 (4)	P1—C11—C20—C19	−178.18 (19)
C3—C4—C5—C6	0.1 (5)	C18—C19—C20—C11	−0.3 (4)
C4—C5—C6—C7	−1.1 (5)	C1—P1—C21—C22	−106.4 (2)
C5—C6—C7—C8	−178.4 (3)	C11—P1—C21—C22	−1.0 (3)
C5—C6—C7—C2	0.6 (5)	C1—P1—C21—C30	79.4 (2)
C3—C2—C7—C6	0.8 (4)	C11—P1—C21—C30	−175.2 (2)
C1—C2—C7—C6	−179.2 (3)	C30—C21—C22—C23	0.5 (4)
C3—C2—C7—C8	179.9 (3)	P1—C21—C22—C23	−173.6 (2)
C1—C2—C7—C8	−0.2 (4)	C21—C22—C23—C24	0.2 (4)
C6—C7—C8—C9	177.9 (3)	C22—C23—C24—C25	−0.8 (4)
C2—C7—C8—C9	−1.2 (4)	C23—C24—C25—C26	−178.7 (2)
C7—C8—C9—C10	1.6 (5)	C23—C24—C25—C30	0.6 (4)
C2—C1—C10—C9	−0.6 (4)	C24—C25—C26—C27	179.6 (3)
P1—C1—C10—C9	−178.7 (2)	C30—C25—C26—C27	0.3 (4)
C8—C9—C10—C1	−0.7 (5)	C25—C26—C27—C28	−0.4 (4)
C1—P1—C11—C20	9.3 (2)	C26—C27—C28—C29	0.5 (4)
C21—P1—C11—C20	−97.2 (2)	C27—C28—C29—C30	−0.6 (5)
C1—P1—C11—C12	−168.88 (18)	C28—C29—C30—C25	0.5 (4)
C21—P1—C11—C12	84.6 (2)	C28—C29—C30—C21	−179.3 (3)
C20—C11—C12—C13	−178.8 (2)	C26—C25—C30—C29	−0.4 (4)
P1—C11—C12—C13	−0.6 (3)	C24—C25—C30—C29	−179.7 (3)
C20—C11—C12—C17	0.2 (3)	C26—C25—C30—C21	179.4 (2)
P1—C11—C12—C17	178.53 (17)	C24—C25—C30—C21	0.1 (4)
C17—C12—C13—C14	−1.0 (4)	C22—C21—C30—C29	179.1 (3)
C11—C12—C13—C14	178.1 (2)	P1—C21—C30—C29	−6.5 (4)
C12—C13—C14—C15	1.0 (4)	C22—C21—C30—C25	−0.6 (4)
C13—C14—C15—C16	−0.2 (4)	P1—C21—C30—C25	173.73 (19)
C14—C15—C16—C17	−0.6 (4)		

### Hydrogen-bond geometry (Å, º)

*Cg*1, *Cg*2, *Cg*3 and *Cg*4 are the centroids of the C2–C7, C12–C17, C25–C30 and C1/C2/C7–C10 rings, respectively.

**Table:**

*D*—H···*A*	*D*—H	H···*A*	*D*···*A*	*D*—H···*A*
C5—H5···Cl1^i^	0.93	2.82	3.512 (4)	132
C18—H18···*Cg*1^ii^	0.93	2.66	3.579 (3)	170
C24—H24···*Cg*2^iii^	0.93	2.51	3.425 (3)	167
C27—H27···*Cg*2^iv^	0.93	2.69	3.612 (3)	170
C8—H8···*Cg*3^v^	0.93	2.79	3.580 (3)	143
C31—H31···*Cg*4^vi^	0.98	2.65	3.618 (6)	172

Symmetry codes: (i) −*x*+2, −*y*+1, −*z*+1; (ii) −*x*+2, −*y*+2, −*z*+1; (iii) *x*−1, *y*, *z*; (iv) −*x*+1, *y*−1/2, −*z*+3/2; (v) *x*, −*y*+3/2, *z*−1/2; (vi) −*x*+1, −*y*+1, −*z*+1.
